# Amino Acid Degrading Enzymes and Autophagy in Cancer Therapy

**DOI:** 10.3389/fphar.2020.582587

**Published:** 2021-01-11

**Authors:** Ziyu Wang, Qinghong Xie, Haifeng Zhou, Min Zhang, Jie Shen, Dianwen Ju

**Affiliations:** ^1^ Department of Pharmacy, Huadong Hospital, Fudan University, Shanghai, China; ^2^ Shanghai Key Laboratory of Clinical Geriatric Medicine, Huadong Hospital, Fudan University, Shanghai, China; ^3^ Department of Biological Medicines & Shanghai Engineering Research Center of Immunotherapeutics, Fudan University School of Pharmacy, Shanghai, China

**Keywords:** autophagy, amino acid degrading enzymes, cancer therapy, L-asparaginase, arginine deiminase, recombinant human arginase, chloroquine

## Abstract

Recently, there has been renewed interest in metabolic therapy for cancer, particularly in amino acid deprivation by enzymes. L-asparaginase was approved for the treatment of acute lymphoblastic leukemia by the U.S. Food and Drug Administration. Arginine deiminase and recombinant human arginase have been developed into clinical trials as potential cancer therapeutic agents for the treatment of arginine-auxotrophic tumors. Moreover, other novel amino acid degrading enzymes, such as glutaminase, methionase, lysine oxidase, phenylalanine ammonia lyase, have been developed for the treatment of malignant cancers. One of the greatest obstacles faced by anticancer drugs is the development of drug resistance, which is reported to be associated with autophagy. Autophagy is an evolutionarily conserved catabolic process that is responsible for the degradation of dysfunctional proteins and organelles. There is a growing body of literature revealing that, in response to metabolism stress, autophagy could be induced by amino acid deprivation. The manipulation of autophagy in combination with amino acid degrading enzymes is actively being investigated as a potential therapeutic approach in preclinical studies. Importantly, shedding light on how autophagy fuels tumor metabolism during amino acid deprivation will enable more potential combinational therapeutic strategies. This study summarizes recent advances, discussing several potential anticancer enzymes, and highlighting the promising combined therapeutic strategy of amino acid degrading enzymes and autophagy modulators in tumors

## Introduction

In recent years, there has been increasing interest in targeting tumor amino acid metabolism as a strategy to treat cancer (Dang et al., 2011; Koppenol et al., 2011). It has been recognized that tumor cells often undergo metabolic reprogramming to support the high metabolic demands that are required for tumorigenesis (Nicholatos et al., 2019; Souder and Anderson, 2019). Cancer cells attempt to utilize various fuel sources to maintain rapid and abnormal proliferation (Vander Heiden and DeBerardinis, 2017). However, certain tumor cells become auxotrophic for specific amino acids, such as asparagine, arginine, and methionine (Cheng et al., 2007; Rytting, 2010; Gao et al., 2019). Therefore, depleting these amino acids by amino acid degrading enzymes inhibits and impairs tumor growth. Whereas, normal cells are kept in good condition due to their capability to synthesize these specific amino acids via endogenous synthesis. The difference between normal and cancer cells in terms of nutritional demand makes tumor tissue vulnerable to certain amino acid deprivation treatments (Fung and Chan, 2017; Dhankhar et al., 2020). Thus, degrading amino acids by enzymes may be an excellent therapeutic approach for the treatment of malignant cancer. L-Asparaginase was the first amino acid degrading enzyme approved by the U.S. Food and Drug Administration (FDA) in 1978, used to treat acute lymphoblastic leukemia (ALL) (Truelove et al., 2013). Since then, many other amino acid depriving enzymes have been developed through preclinical or clinical evaluations (Stasyk et al., 2015; Fernandes et al., 2017).

There is a growing body of literature indicating that cellular metabolism stress, for example, nutrient deprivation, could induce macroautophagy (Xie et al., 2015; Fulop et al., 2018; Nacarelli et al., 2018). Macroautophagy is an evolutionarily conserved catabolic process in which damaged cellular organelles and proteins are engulfed into double-membraned vesicles named autophagosomes, and then delivered to lysosomes for degradation (Nishida et al., 2009; Honda et al., 2014). Besides macroautophagy, there are other categories of autophagy, including macroautophagy, microautophagy, and chaperone-mediated autophagy (Mony et al., 2016). Macroautophagy (hereafter referred to as autophagy) plays a crucial role not only in normal cells and tissues but also in pathological environments. Amino acid starvation initiates autophagy by inhibiting the mammalian target of rapamycin (mTOR) and activating adenosine 5′-monophosphate (AMP)-activated protein kinase (AMPK). In the initial stage, the ULK1 and VPS34 complexes are most essential in recruiting and assembling other components that are needed for autophagy. There are more autophagy-related genes (ATGs) involved in the whole process of autophagy (Hsu et al., 2018; Chang, 2020; Gu et al., 2020). A large number of studies have been published on the complicated and context-dependent role of autophagy in cancer (White et al., 2015; Levy et al., 2017). Although autophagy served as a double-edged sword in the carcinogenesis, progression, treatment, and resistance of tumors (White, 2015; Limpert et al., 2018; Monkkonen and Debnath, 2018), most studies have suggested that autophagy is vital to promote tumor growth and survival. Nowadays, blocking autophagy as a potential anticancer therapy is currently undergoing clinical trials (Bortnik and Gorski, 2017; Chude and Amaravadi, 2017). Autophagy serves a cytoprotective role in cancer through its capability to support cancer metabolism. Given that autophagy can degrade various substrates, it is not surprising that autophagy provides cancer cells with abundant metabolic plasticity, for example, degradation of protein or peptide into amino acid could fuel the tricarboxylic acid (TCA) cycle (Kimmelman and White, 2017).

Importantly, the combination of amino acid degrading enzymes and autophagy regulators has been demonstrated to show marvelous synergistic anticancer effects in preclinical and clinical studies (Kim et al., 2009b; Zeng et al., 2013; Song et al., 2015). This review will highlight recent advances in the development of amino acid depriving enzymes and the combinational employment of autophagy regulators and enzymes which have been successfully used as potential therapeutic approaches in the therapy of cancer.

### L-Asparaginase and Autophagy

L-asparaginase, the first bacterial enzyme approved for cancer therapy, hydrolyzes L-asparagine (ASN) and yields aspartic acid and ammonia (Pieters et al., 2011). ASN plays an important role in glycoproteins biosynthesis, regulating the functions of the immune and nervous systems (Wu, 2013; Knott et al., 2018). Importantly, several types of tumors, particularly leukemia cells, cannot synthesize ASN due to lack of or low expression of asparagine synthetase (Willems et al., 2013), which renders the tumors sensitive to asparaginase. The first commercial drug of L-asparaginase is a native *E. coli*-derived asparaginase, an indication of which is ALL. Although L-asparaginase derived from *E. coli* achieved great therapeutic improvements, it is subject to hypersensitivity and other toxicities, such as hepatic and renal dysfunction (Spiers and Wade, 1979; Salzer et al., 2014). A more stable and efficient form of L-asparaginase derived from *E. coli* was PEGylated to reduce the allergy to foreign proteins and prolong half-life (Dinndorf et al., 2007). Nowadays, L-asparaginase derived from *E. coli* has been applied as first-line therapy and L-asparaginase derived from *Erwinia chrysanthemi* has been used for the treatment of ALL patients when hypersensitivity to *E. coli*-derived L-asparaginase happens (Keating, 2013). Apart from hypersensitivity, glutamine depletion is another clinical problem of L-asparaginase due to its dual asparaginase and glutaminase activity, which can cause hepatotoxicity, thrombotic complication, and neurotoxicity (Reinert et al., 2006). Researchers have explored solutions by modifying L-asparaginase via replacing amino acid residues (Derst et al., 2000).

**FIGURE 1:**
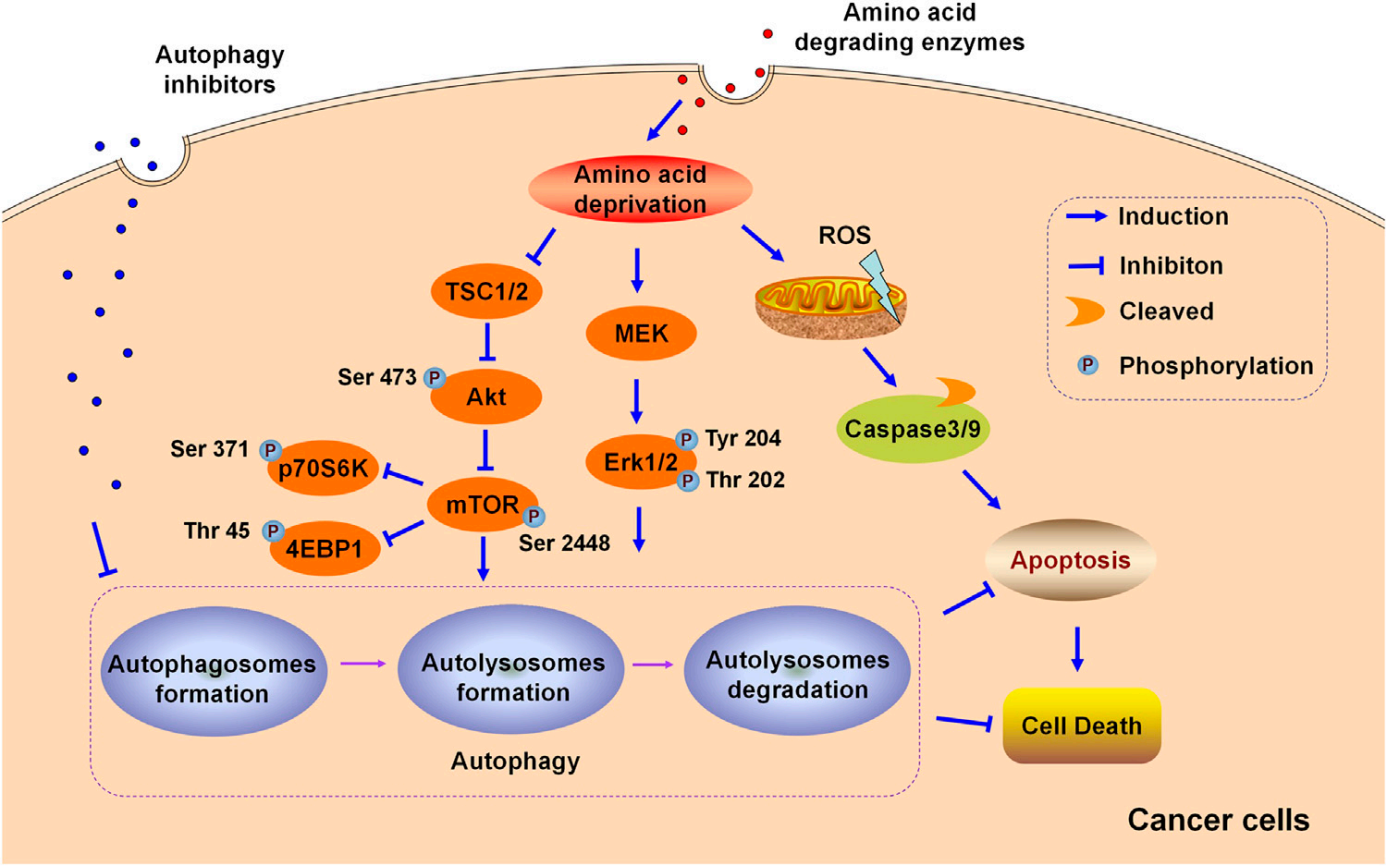
Schematic illustration of the cytoprotective role of autophagy in the cytotoxicity induced by amino acid degrading enzymes (L-Asparaginase, ADI, and rhArg) in cancer cells.

One of the greatest obstacles faced by L-asparaginase in clinical applications is the development of drug resistance. We reported that L-asparaginase not only induced caspase 3-dependent apoptosis but also triggered obvious autophagy in chronic myeloid leukemia (CML) cells, accompanied by inhibition of Akt/mTOR and activation of the ERK signaling pathway (Song et al., 2015), as illustrated in Figure 1. The blocking of autophagy by LY294002, chloroquine (CQ), and quinacrine enhanced apoptosis is triggered by L-asparaginase, suggesting the pro-survival role of autophagy in L-asparaginase-treated CML cells. Moreover, the ROS-p53 feedback loop played an important role in the combinational treatment of L-asparaginase and CQ. In addition to CML, ALL, glioblastoma, laryngeal squamous cell carcinoma, and pulmonary adenocarcinoma showed sensitivity to L-asparaginase, and autophagy was demonstrated to be activated through autophagosomes formation and the conversion of cytoplasmic LC3-I to membranal LC3-II (Zhang et al., 2016; Chen et al., 2017; Ji et al., 2017; Takahashi et al., 2017; Polak et al., 2019). Moreover, the combination of CQ and L-asparaginase significantly enhanced the antitumor effect of L-asparaginase. Based on the studies mentioned, autophagy played a cytoprotective role in most cancer therapy of L-asparaginase, which indicated that both targeting asparagine metabolism and autophagy was a new promising therapeutic strategy for malignant tumors. More studies and evaluations of the combinational treatment of amino acid degrading enzymes and autophagy regulators are listed in Table 1.

**TABLE 1:** Current developments of the combination of amino acid degrading enzymes and autophagy inhibitors in pre-clinical studies.

Enzymes	Combined treatment	Types of cancer	Level proof-of concept	References
L-asparaginase	L-asparaginase + CQ	ALL	*In vitro* and *in vivo*	Takahashi et al. (2017), Polak et al. (2019)
	L- asparaginase + CQ/LY294002/quinacrine	CML	*In vitro*	Song et al. (2015)
	L-asparaginase + CQ/LY294002	Glioblastoma	*In vitro* and *in vivo*	Chen et al. (2017)
	L-asparaginase + CQ	Laryngeal squamous cell carcinoma	*In vitro*	Ji et al. (2017)
	L-asparaginase + CQ	Pulmonary adenocarcinoma	*In vitro*	Zhang et al. (2016)
Arginine deiminase	Arginine deiminase + CQ	Lymphoma	*In vitro*	Delage et al. (2012)
	Arginine deiminase + CQ/siBeclin1	Melanoma	*In vitro*	Savaraj et al. (2010)
	Arginine deiminase + CQ/siBeclin1	Prostate cancer	*In vitro*	Kim et al. (2009b)
Arginase I	rhArg + 3MA/CQ/siBeclin1	Triple-negative breast cancer	*In vitro*	Wang et al. (2014)
	rhArg + 3MA/CQ	Leukemia	*In vitro*	Li et al. (2016)
	rhArg + CQ	Ovarian cancer	*In vitro*	Nasreddine et al. (2020)
	rhArg + CQ/siAtg5	Melanoma	*In vitro*	Wang et al. (2014)
	rhArg + 3MA/CQ/siBeclin 1/siAtg5	Non-Hodgkin’s lymphoma	*In vitro*	Zeng et al. (2013)
	rhArg + CQ/Baf A1	Laryngeal squamous cell carcinoma	*In vitro*	Lin et al. (2015)
	rhArg + CQ/LY294002	Non-small cell lung cancer	*In vitro* and *in vivo*	Shen et al. (2017)

It is noteworthy that there is a close relationship between autophagy and the immune system (Gonzalo and Coll-Bonfill, 2019), which is vital for efficient cancer therapy (Jin et al., 2017; Yamamoto et al., 2020). L-asparaginase is demonstrated to not only suppress autophagy in macrophages but also inhibit phagocytosis, MHC-II expression, secretion of cytokine IL-6, and TNF-α in activated macrophages. Importantly, activating autophagy could overcome the immune suppression triggered by L-asparaginase in macrophages (Song et al., 2017).

## Arginine Deiminase and Autophagy

Arginine is a semi-essential amino acid that cannot be adequately supplied by endogenous biosynthesis. Arginine metabolism is complicated, as arginine plays an essential role in several biological functions, such as precursors for cell signaling molecules as well as intermediates of the urea cycle and TCA cycle (Shen and Shen, 2006; Ni et al., 2008). Arginine can be produced through sucargininosuccinate synthetase (ASS), ornithine transcarbamylase (OTC), and argininosuccinate lyase in the urea cycle. ASS and OTC are the key enzymes for arginine synthesis (Fultang et al., 2020).

Arginine deiminase (ADI), an arginine-depleting enzyme, is regarded as a novel anticancer candidate (Synakiewicz et al., 2014) and hydrolyzes arginine into citrulline and ammonia. For ADI, ASS-deficient tumors are arginine-auxotrophic and its efficiency is highly dependent on the ASS deficiency of cancer patients (Lam et al., 2009). ASS silencing in cholangiocarcinoma cells (Roeksomtawin et al., 2018) and glioblastoma multiforme cells (Przystal et al., 2018) significantly enhanced their sensitivity to ADI-PEG20 treatment. It is reported that ADI-PEG20 significantly reduced tumor growth in ASS^low^ sarcoma models. However, significantly increased expression of ASS was observed in tumor tissue over time (Delage et al., 2012; Bean et al., 2016). In randomized clinical trials, neutralizing antibodies against ADI-PEG20 and enhanced ASS expression was found in the latter treatment, which caused drug resistance (Szlosarek et al., 2017). Pegylation significantly reduces antigenicity of ADI and ADI-PEG20 has been used in clinical trials in patients with hepatocellular carcinoma (HCC) (in phase III), melanoma (in phase I/II), small cell lung cancer (SCLC) (in phase II), mesothelioma (in phase I/II) and other arginine-auxotrophic advanced tumors.

In addition to neutralizing antibodies and enhanced ASS expression, autophagy is reported to be associated with ADI-resistance. Arginine deprivation by ADI could induce obvious autophagy and autophagy inhibitors potentiated the cytotoxicity of ADI-PEG20 in lymphoma cells, indicating the cytoprotective role of autophagy triggered by ADI-PEG20 in lymphoma. Furthermore, blocking autophagy by CQ or silencing autophagy-related Beclin1 mRNA accelerated and enhanced the antitumor effect of ADI in melanoma (Savaraj et al., 2010) and prostate cancer (Kim et al., 2009a; Kim et al., 2009b), suggesting that both targeting autophagy and arginine metabolism may provide novel potential avenues for cancer therapy. ADI activated MEK and ERK signaling pathways but suppressed the Akt/mTOR pathway in melanoma cells, as shown in Figure 1. In general, Akt/mTOR and ERK signaling pathways are vital in manipulating autophagy in eukaryotic cells (Dai et al., 2019; Farias Quipildor et al., 2019). Nutritional starvation triggers autophagy via inhibiting mTOR, which is a vital negative regulator of autophagy. The ERK signaling pathway is documented to regulate the induction of autophagy by interacting with LC3 and the expression of autophagy as well as lysosomal genes.

It was observed that depriving arginine by ADI triggered a cytotoxic excessive autophagy which contributed to cell death in ASS-deficient prostate cancer cells. Representative micrographs exhibited atypical autophagy with large autophagosomes formation, nucleus membrane rupture, and DNA/chromatin leakage was captured by autophagosomes, which was referred to as chromatin autophagy (Changou et al., 2014; Li et al., 2019). ADI triggered regular-sized autophagosomes during the first 24 h and giant autophagosomes after 48 h in prostate cancer cells. Moreover, ADI triggered mitochondrial dysfunction, for example, mitochondrial membrane potential depolarization (Kung et al., 2015).

### Arginase and Autophagy

Another arginine degrading enzyme used for cancer therapy is arginase I. Previous studies have suggested that cancers with deficiencies in either ASS and/or OTC expression are sensitive to arginine-auxotrophy induced by arginase I (Cheng et al., 2007; Tsui et al., 2009). Recombinant human arginase I (rhArg) is a modified enzyme, which contains cobalt (II) ion or manganese (II) ion (Cheng et al., 2007; Lam et al., 2009; Yau et al., 2013). During a preclinical evaluation, rhArg exhibited significant antitumor activity in many ASS and/or OTC deficient cancer cells, namely HCC (Cheng et al., 2007), melanoma (Wang et al., 2014b), breast cancer (Leung et al., 2019), keratinocytic carcinoma (Bobak et al., 2010), SCLC (Xu et al., 2018) and Merkel cell carcinoma (Agnello et al., 2020). Pegylated rhArg has a remarkable advantage over the native arginase I on account of the extended half-life, from several hours to 72–96 h, due to its enhanced stability (Lam et al., 2011). According to the records on ClinicalTrials.gov, PEG-rhArg has been studied in clinical trials for a variety of malignant cancers, including HCC, pediatric AML, pediatric ALL, and prostate adenocarcinoma.

Arginase I is mainly expressed in the liver. Blocking autophagy by liver-specific deletion of the important autophagy genes Atg7 and Atg5, which generated circulating arginase I and inhibited tumor growth and identifies a metabolic vulnerability of cancer. Moreover, supplementation with arginine in Atg7-deficient mice model partially relieved arginine reduction and tumor growth inhibition. Whole-body deletion of Atg7 in a mice model triggered a bigger regression of KRAS-driven tumors than the knockdown of cancer-specific autophagy, suggesting that basal autophagy facilitates tumor growth (Poillet-Perez et al., 2018). Previously, we reported that rhArg suppressed cell growth of triple-negative breast cancer (TNBC), which lacks an effective druggable target, resulting in poor prognosis. Also, autophagic flux was observed in TNBC cells. Blocking autophagy by CQ, 3-MA and silencing Beclin1 enhanced the antitumor effect of rhArg in TNBC (Wang et al., 2014a). Until now, rhArg was also found to have an inhibitory effect on melanoma cells (Wang et al., 2014b), non-Hodgkin’s lymphoma cells (Zeng et al., 2013), laryngeal squamous cell carcinoma cells (Lin et al., 2015), leukemia cells (Li et al., 2016), ovarian cancer cells (Nasreddine et al., 2020), and non-small-cell lung cancer (NSCLC) cells (Shen et al., 2017). Autophagy inhibitors enhanced the antitumor effect of rhArg in these tumors, indicating that autophagy is pro-survival in the treatment of cancer (as shown in Table 1). Notably, arginase I was reported to contribute to tumor-driven immune suppression which is a major obstacle for the immunotherapy of cancer (Czystowska-Kuzmicz et al., 2019; Wang et al., 2019).

## Other Amino Acid Enzymes and Autophagy

Apart from asparaginase, arginine deiminase, arginase, some other amino acid enzymes have been recently developed for cancer therapy, including methionase, lysine oxidase, phenylalanine ammonia lyase, and glutaminase. These amino acid degrading enzymes and their related autophagy studies are relatively fewer than the three enzymes discussed above.

Glutaminase is a vital enzyme that breaks down glutamine into glutamate. Glutaminase is not regarded as a potential drug for cancer therapy, but, instead, as a druggable target (Masisi et al., 2020). Cancers with high glutaminase expression are related to poor prognosis. Recently, the strategy of cancer therapy in glutamine metabolism inhibition has begun to concentrate on glutamine deprivation, glutaminase blocking, and membrane glutamine transporter inhibition (Chiu et al., 2014; Gross et al., 2014; Lee et al., 2014; Song et al., 2018). Among glutaminase inhibitors, CB-839, one of the most successful drug candidates, is under clinical trials for NSCLC, melanoma, and leukemia (NCT03965845, NCT02771626, and NCT02071927 respectively). It was reported that glutamine deprivation was synthetically lethal for autophagy inhibition in colorectal cancer (Li et al., 2017). Autophagy is an essential process that provides glutamine for anaplerosis of the TCA cycle in pancreatic ductal adenocarcinoma. Therefore, targeting glutamine metabolism and autophagy simultaneously to completely inhibit glutamine uptake offers a novel therapeutic approach for treating refractory cancers (Seo et al., 2016).

Methionase, also named L-Methionine-γ-lyase, converts methionine into ammonia, α-ketobutyrate, and methanethiol (Cellarier et al., 2003; Thivat et al., 2007; Ho et al., 2011). Methionine-dependent cancer cannot generate or generate low levels of methionine. Methionase was regarded as a potential anticancer candidate for Lewis lung, human colon carcinoma (Tan et al., 1998), neuroblastoma (Hu and Cheung, 2009), and glioblastoma (Kokkinakis et al., 2001). PEGylated recombinant methionase has been developed into phase I clinical trials, in which recombinant methionase showed no significant toxicity (Tan et al., 1996; Tan et al., 1997). However, the antitumor activity of PEGylated recombinant methionase was not reported. Notably, methionine acts as a signal for amino acid which could suppress autophagy induced by nitrogen starvation via methylation of PP2A (a protein phosphatase enzyme), also depleting methionine and cystine induced autophagy and suppressed tumor growth in glioma cells *in vivo*.

Lysine oxidase, one of the most studied amino acid oxidases, showed considerable cytotoxicity against a wide variety of cancers, including leukemia, colorectal adenocarcinoma, prostate cancer, pheochromocytoma (Pokrovsky et al., 2013; Lukasheva et al., 2015). The short half-life of lysine oxidase restricted its development and commercialization (Krupyanko et al., 2017). Moreover, a few studies have shown that lysine oxidase supports the growth of some tumors (Wang et al., 2016), which makes the role of lysine oxidase in antitumor therapy controversial and, therefore, demands more preclinical data.

Phenylalanine ammonia lyase converts phenylalanine to trans-cinnamic acid and ammonia. Like other enzymes. The antitumor mechanism of phenylalanine ammonia lyase is associated with a reduced level and disability of synthesis of phenylalanine. Phenylalanine ammonia lyase showed to be effective against colorectal cancer *in vivo* (Yang et al., 2019) and leukemic lymphoblasts *in vitro* (Stith et al., 1973).

## Conclusion

There exist several advantages of amino acid degrading enzymes over conventional anticancer therapeutics. Firstly, amino acid enzymes have strong effects against specific amino acid auxotrophic tumors. Secondly, the side effect pattern of the enzymes is unique, which is significant for drug combinational therapy. Lastly, there exist key synthetases as biomarkers to forecast the therapeutic effect (Timosenko et al., 2017; Pokrovsky et al., 2019). Clinical trials of amino acid-degrading enzymes have shown that enzyme treatment is a safe and effective therapeutic approach. Despite the advantages of amino acid in depleting enzymes, a few weaknesses still affect clinical applications. The high immunogenicity and shorter half-life may be the greatest obstacles in the development of drugs (Schiffmann et al., 2019; Thisted et al., 2019). Chemical modification, construction of fusion protein, and encapsulation of enzymes are some of the existing solutions to overcome those obstacles and increase the bioavailability of amino acid degrading enzymes (Veronese, 2001; Li et al., 2007; Chen and Zeng, 2016; Bilal et al., 2018; Sinha and Shukla, 2019).

Recently, both targeting autophagy and amino acid metabolism have entered into clinical studies on the basis of preclinical experiments (as shown in Table 1) and synergistic drug effects in cancer therapy. Combinational therapy is a great opportunity for cancer patients. Although the context-dependent role of autophagy during tumor treatment has attracted great attention, amino acid degrading enzyme induced pro-survival autophagy in the majority of tumors. Therefore, manipulating autophagy provides a chance to make a tumor more sensitive to subsequent therapeutics. Among them, CQ is one of the most used autophagy inhibitors. CQ inhibits autophagosome fusing with lysosome, and significantly improves the expression level of LC3-II. Furthermore, there is a growing body of literature that recognizes the importance of potential applications of autophagy related proteins, including LC3, ATG7, ATG5, Beclin1, and SH3GLB1, as prognostic biomarkers in some tumors, like glioma, breast cancer, and colon cancer (Park et al., 2013; Lebovitz et al., 2015). Under the right conditions, in the future, a co-targeting autophagy and amino acid metabolism may become a potential cancer therapy.

Despite the advances mentioned in this study, patients still have a poor prognosis. Hence, further studies are required to provide a deeper understanding of the underlying molecular mechanisms and more clinical trials are needed to collect evidence-based data with respect to the efficacy and safety of these therapeutics.

## Author Contributions

ZW made the draft. QX, JS, and ZS revised the manuscript. HZ and MZ analyzed the scientific literature. DJ designed the study and revised the manuscript.

## Funding

This work was supported by grants from the National Natural Science Foundation of China (No. 81773620, 31872746), the Shanghai Sailing Program (17YF1405100) and Key Innovative Team of Shanghai Top-Level University Capacity Building in Clinical Pharmacy and Regulatory Science at Shanghai Medical College, Fudan University (HJW-R-2019-66-19).

## Conflict of Interest

The authors declare that the research was conducted in the absence of any commercial or financial relationships that could be construed as a potential conflict of interest.

## Abbreviations

This article was submitted to Pharmacology of Anti-Cancer Drugs, a section of the journal Frontiers in Pharmacology

## Abbreviations

3MA, 3-methyladenine; ALL, acute lymphoblastic leukemia; AML, acute myeloid leukemia; Baf A1, bafilomycin A1; CQ, chloroquine; rhArg, recombinant human arginase I; siAtg5, siRNA targeting Atg5; siBeclin, siRNA targeting Beclin1.
